# Discovering novel germline genetic variants linked to severe fluoropyrimidine-related toxicity in- and outside *DPYD*

**DOI:** 10.1186/s13073-024-01354-z

**Published:** 2024-08-15

**Authors:** Jonathan E. Knikman, Qinglian Zhai, Carin A. T. C. Lunenburg, Linda M. Henricks, Stefan Böhringer, Maaike van der Lee, Femke M. de Man, Steven M. Offer, Shikshya Shrestha, Geert-Jan Creemers, Arnold Baars, Vincent O. Dezentjé, Alexander L. T. Imholz, Frank J. F. Jeurissen, Johanna E. A. Portielje, Rob L. H. Jansen, Paul Hamberg, Helga J. Droogendijk, Miriam Koopman, Peter Nieboer, Marlène H. W. van de Poel, Caroline M. P. W. Mandigers, Ron H. N. van Schaik, Hans Gelderblom, Ron H. J. Mathijssen, Jan H. M. Schellens, Annemieke Cats, Henk-Jan Guchelaar, Jesse J. Swen

**Affiliations:** 1 Division of Pharmacology, The Netherlands Cancer Institute, Amsterdam, The Netherlands; 2 Department of Clinical Pharmacy and Toxicology, Leiden University Medical Center, Leiden, The Netherlands; 3 Department of Medical Oncology, Leiden University Medical Center, Leiden, The Netherlands; 4 Department of Clinical Chemistry and Laboratory Medicine, Leiden University Medical Center, Leiden, The Netherlands; 5 Department of Biomedical Data Sciences, Leiden University Medical Center, Leiden, The Netherlands; 6 Department of Medical Oncology, Erasmus MC Cancer Institute, Erasmus University Medical Center, Rotterdam, The Netherlands; 7 Department of Pathology, Carver College of Medicine, University of Iowa, Iowa City, IA USA; 8 Holden Comprehensive Cancer Center, University of Iowa, Iowa City, IA USA; 9 Mayo Clinic Graduate School of Biomedical Sciences, Mayo Clinic, Rochester, MN USA; 10 Division of Pulmonary and Critical Care Medicine, Brigham and Women’s Hospital, Harvard Medical School, Boston, MA USA; 11 Department of Medical Oncology, Catharina Hospital, Eindhoven, The Netherlands; 12 Department of Internal Medicine, Hospital Gelderse Vallei, Ede, The Netherlands; 13 Department of Internal Medicine, Reinier de Graaf Hospital, Delft, The Netherlands; 14 Division of Medical Oncology, The Netherlands Cancer Institute, Amsterdam, The Netherlands; 15 Department of Internal Medicine, Deventer Hospital, Deventer, The Netherlands; 16 Department of Internal Medicine, Haaglanden Medical Center, The Hague, The Netherlands; 17 Department of Internal Medicine, Maastricht University Medical Center, Maastricht, The Netherlands; 18 Department of Internal Medicine, Franciscus Gasthuis en Vlietland, Rotterdam, The Netherlands; 19 Department of Internal Medicine, Bravis Hospital, Roosendaal, The Netherlands; 20 Department of Medical Oncology, University Medical Center Utrecht, Utrecht, The Netherlands; 21 Department of Internal Medicine, Wilhelmina Hospital Assen, Assen, The Netherlands; 22 Department of Internal Medicine, Laurentius Hospital, Roermond, The Netherlands; 23 Department of Internal Medicine, Canisius-Wilhelmina Hospital, Nijmegen, The Netherlands; 24 Department of Clinical Chemistry, Erasmus University Medical Center, Rotterdam, Netherlands; 25 Department of Pharmaceutical Sciences, Utrecht University, Utrecht, The Netherlands; 26 Department of Gastroenterology and Hepatology, Division of Medical Oncology, The Netherlands Cancer Institute, Amsterdam, The Netherlands

**Keywords:** Fluoropyrimidines, Pharmacogenetics, Personalized medicine, *DPYD*, Dihydropyrimidine dehydrogenase

## Abstract

**Background:**

The Alpe-DPD study (NCT02324452) demonstrated that prospective genotyping and dose-individualization using four alleles in *DPYD* (DPYD*2A/rs3918290, c.1236G > A/rs75017182, c.2846A > T/rs67376798 and c.1679 T > G/rs56038477) can mitigate the risk of severe fluoropyrimidine toxicity. However, this could not prevent all toxicities. The goal of this study was to identify additional genetic variants, both inside and outside *DPYD*, that may contribute to fluoropyrimidine toxicity.

**Methods:**

Biospecimens and data from the Alpe-DPD study were used. Exon sequencing was performed to identify risk variants inside *DPYD*. In silico and in vitro analyses were used to classify *DPYD* variants. A genome-wide association study (GWAS) with severe fluoropyrimidine-related toxicity was performed to identify variants outside *DPYD*. Association with severe toxicity was assessed using matched-pair analyses for the exon sequencing and logistic, Cox, and ordinal regression analyses for GWAS.

**Results:**

Twenty-four non-synonymous, frameshift, and splice site *DPYD* variants were detected in ten of 986 patients. Seven of these variants (c.1670C > T, c.1913 T > C, c.1925 T > C, c.506delC, c.731A > C, c.1740 + 1G > T, c.763 − 2A > G) were predicted to be deleterious. The carriers of either of these variants showed a trend towards a 2.14-fold (95% CI, 0.41–11.3, *P* = 0.388) increased risk of severe toxicity compared to matched controls (*N* = 30). After GWAS of 942 patients, no individual single nucleotide polymorphisms achieved genome-wide significance (*P* ≤ 5 × 10^−8^), however, five variants were suggestive of association (*P* < 5 × 10^−6^) with severe toxicity.

**Conclusions:**

Results from *DPYD* exon sequencing and GWAS analysis did not identify additional genetic variants associated with severe toxicity, which suggests that testing for single markers at a population level currently has limited clinical value. Identifying additional variants on an individual level is still promising to explain fluoropyrimidine-related severe toxicity. In addition, studies with larger samples sizes, in more diverse cohorts are needed to identify potential clinically relevant genetic variants related to severe fluoropyrimidine toxicity.

**Supplementary Information:**

The online version contains supplementary material available at 10.1186/s13073-024-01354-z.

## Background

Fluoropyrimidines, including 5-fluorouracil (5-FU) and capecitabine, represent the backbone of chemotherapeutic regimens used in the treatment of solid tumours, such as gastroesophageal, colorectal, and breast cancer. Depending on the treatment regimen administered, severe fluoropyrimidine-induced toxicity affects approximately 35% of recipients and can be lethal in up to 1% of the patients [1]. Common fluoropyrimidine-induced adverse events include diarrhoea, mucositis, hand-foot syndrome, and myelosuppression. An increased risk for the development of fluoropyrimidine-induced toxicity exists in patients with a deficiency of dihydropyrimidine dehydrogenase (DPD), an enzyme that is encoded by the *DPYD* gene and responsible for catalysing 5-FU degradation into inactive metabolites [2]. Both DPD activity and genetic variants in *DPYD* have been widely investigated and partially explain severe fluoropyrimidine-induced toxicity. Previous studies and meta-analyses have shown a strong association between four *DPYD* variants (c.1905 + 1G > A/rs3918290, c.1236G > A/rs56038477, c.2846A > T/rs67376798, and c.1679 T > G/rs55886062) and severe fluoropyrimidine-induced toxicity [3, 4]. Recently, we showed that patients’ safety indeed improved following fluoropyrimidine dose individualization based on *DPYD*-genotyping of the four *DPYD* variants mentioned above [5]. Consistent with these findings, the European Medicine Agency (EMA) recently recommended that all patients scheduled for fluoropyrimidine treatment should be tested for DPD deficiency before the start of treatment [6].

Despite the recognition of the importance of the abovementioned four variants in reducing toxicity, approximately 25% of *DPYD* wild-type patients still experienced severe fluoropyrimidine-induced toxicity [5]. This suggests that additional factors, including other *DPYD* genetic variants and/or variants affecting other genes involved in fluoropyrimidine metabolism, may contribute to toxicity. Indeed, low-frequency germline variants (minor allele frequencies (MAFs) < 1%) may explain approximately 30–40% of inter-individual functional variability in pharmacogenes [7]. However, the effect of these low-frequency variants in *DPYD* has not been assessed comprehensively in fluoropyrimidine-treated patient populations.

In the present study, we sought to identify potential biomarkers of severe fluoropyrimidine toxicity risk in a patient population that did not carry any of the four well-characterized risk alleles in *DPYD*. To accomplish this goal, we used complementary approaches for genotyping that included both targeted sequencing of the exon-coding region for *DPYD* and genome-wide association study (GWAS) in cancer patients treated with fluoropyrimidines.

## Methods

### Patients

Clinical data including baseline characteristics and toxicity data were derived from patients included in the Alpe-DPD study (clinicaltrial.gov identifier NCT02324452) [5]. The design, study population, and results of the Alpe-DPD study have been previously published [5]. In brief, adult patients (≥ 18 years) with cancer who were intended to start treatment with fluoropyrimidine-based therapy in 17 hospitals in the Netherlands were included. Patients with all tumour types for which fluoropyrimidine treatment was indicated were eligible. Prospective genotyping for DPYD*2A, c.2846A > T, c.1679 T > G, and c.1236G > A was performed. Heterozygous *DPYD* variant carriers received an initial dose reduction of 50% (DPYD*2A and c.1679 T > G) or 25% (c.1236G > A and c.2846A > T). *DPYD* wildtype patients were treated according to standard-of-care. The primary endpoint of the study was the frequency of severe fluoropyrimidine-related toxicity across the entire treatment duration. All patients (*N* = 1181 of which 1103 were evaluable) signed informed consent before inclusion in the study, which included approval for the use of clinical data and remaining material (whole blood samples taken before the start of the chemotherapy in a 4-ml EDTA tube) to perform *DPYD* sequencing and GWAS. In- and exclusion criteria can be found in the Additional file 1. The baseline characteristics of the cohort can be found in Table 1. Toxicity was graded according to the National Cancer Institute common terminology criteria for adverse events (CTC-AE; version 4.03) and severe toxicity was defined as CTC-AE grade ≥ 3 [8]. Only the highest graded adverse events classified as possible, probable, or definite related to fluoropyrimidines were included in the analyses [5].

**Table 1:** Patient characteristics

Characteristic	Evaluable patients (Alpe-DPD cohort) (*N* = 1103)	GWAS cohort (*N* = 599)
Gender		
*Male*	593 (54%)	319 (53%)
*Female*	510 (46%)	280 (47%)
Age in years, median, (IQR)	64 (56–71)	64 (57–71)
Ancestry		
*White*	1048 (95%)	573 (96%)
*Black*	19 (2%)	14 (2%)
*Asian*	24 (2%)	9 (2%)
*Other*^*a*^	12 (1%)	3 (< 1%)
Tumour type		
*Non-metastatic colorectal cancer*	472 (43%)	265 (44%)
*Metastatic colorectal cancer*	232 (21%)	114 (19%)
*Breast cancer*	141 (13%)	75 (13%)
*Gastric cancer*	63 (6%)	32 (5%)
*Other*^*b*^	195 (18%)	113 (19%)
Type of treatment regimen		
*Capecitabine monotherapy (*± *bevacizumab)*	205 (19%)	102 (17%)
*Capecitabine* + *radiotherapy (*± *mitomycin)*	264 (24%)	172 (29%)
*Capecitabine* + *oxaliplatin (*± *bevacizumab)*	374 (34%)	179 (30%)
*Capecitabine* + *other anticancer drugs*	72 (7%)	41 (7%)
*Fluorouracil monotherapy*	2 (< 1%)	-
*Fluorouracil* + *radiotherapy (*± *mitomycin)*	63 (6%)	43 (7%)
*Fluorouracil* + *oxaliplatin* + *folinic acid (*± *bevacizumab)*	43 (4%)	18 (3%)
*Fluorouracil* + *other anticancer drugs*	80 (7%)	44 (7%)
BSA, median (IQR)	1.9 (1.8–2.1)	1.9 (1.8–2.1)
WHO performance status		
*0*	554 (50%)	317 (53%)
*1*	448 (41%)	241 (40%)
*2*	42 (4%)	21 (4%)
*Not specified*^*c*^	59 (5%)	20 (4%)
Number of treatment cycles, median (IQR)	3 (1–8)	3 (1–7)

*Abbreviations: IQR*, interquartile range; *BSA*, body surface area; *DPD*, dihydropyrimidine dehydrogenase; *DPYD*, gene encoding dihydropyrimidine dehydrogenase; *WHO*, World Health Organization

Patient characteristics of evaluable patients (*N* = 1103) and the patients included in the primary analysis of the GWAS (*N* = 599). Data are *n* (%) or median (IQR)

^a^Other ethnic origins included Hispanic descent, mixed racial parentage, and unknown ethnic origin

^b^Other tumour types included anal cancer, oesophageal cancer, head and neck cancer, pancreatic cancer, bladder cancer, vulvar cancer, unknown primary tumours, and rare tumour types

^c^WHO performance status was not specified for these patients, but was either 0, 1, or 2, as required by the study inclusion criteria

### *DPYD* sequencing

#### Genotyping

Targeted DNA sequencing was performed for specimens with adequate DNA (*N* = 1103) extracted from whole blood samples (4 ml in an EDTA tube). Sequencing libraries were generated using Access Array chemistry (Fluidigm, South San Francisco, CA) as previously described, with modifications [9]. Custom primer panels were designed to cover all 23 exons of the *DPYD* gene and the intronic region containing rs75017182, the causal single nucleotide polymorphisms (SNP) in perfect linkage with c.1236G > A/HapB3. Target amplification and sample indexing were performed using Juno Targeted Sequencing LP 192.24 Integrated Fluidic Circuits (IFCs) on a Juno instrument (Fluidigm). Indexed sequencing libraries from 2 IFCs were pooled, and paired-end sequencing was performed using an Illumina HiSeq 4000 in the Mayo Clinic Cancer Center Genome Analysis Core. Patient sequence data were demultiplexed using barcode sequences added during library preparation. Adapter and region-specific primer sequences were pruned, and reads were aligned to targeted regions of the hg38 human reference genome using BWA-MEM. Variants were identified using GATK HaplotypeCaller. A QUAL score of ≥ 500 across the population of samples tested was used as a threshold for variant inclusion in subsequent analyses. The presence of toxicity-associated variants (DPYD*2A, c.1236G > A, c.2846A > T, or c.1679 T > G) was confirmed using previous genotyping data [5]. The genotypes for additional rare variants with allele frequencies less than 1% in the study population were confirmed in carriers by Sanger sequencing of the relevant exon at the Mayo Clinic Cancer Center Genomics Analysis Core using methods that have been previously described [9].

#### Variant classification

**Fig. 1:**
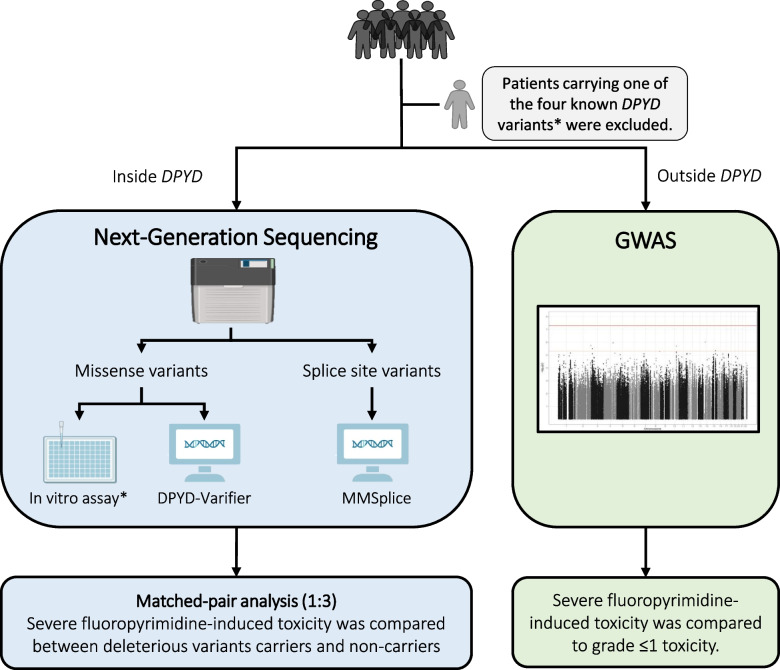
Study design. Severe fluoropyrimidine-related toxicity was defined as CTC-AE grade ≥ 3. *The four known DPYD variants are DPYD*2A, c.1236G > A, c.2846A > T, and c.1679 T > G

In this study, different in vitro and in silico approaches were used to assess the potential effect of identified *DPYD* variants (Fig. 1). Missense variants were evaluated using a previously published in vitro expression system in HEK293T/c17 cells. If available, results for variants were reused, otherwise, novel variants were expressed in the in vitro system [10, 11]. Detailed primer sequences used to generate the expression plasmids for selected variants are reported in the Additional file 1: Table S1. The *DPYD*-Varifier, a *DPYD*-specific in silico prediction tool applied for eligible variants [12]. Results of the in vitro assay are used as the final decision of the variant function if the *DPYD*-Varifier has an inconsistent prediction [12]. Frameshift variants were considered deleterious based on previous findings [11]. The potential impact of splice site variants was predicted using MMsplice, a modelling-based tool to predict genetic variation effects on splicing [13].

#### Statistical analysis

After *DPYD* sequencing, all patients who carried at least one predicted deleterious variant were matched with wild-type controls identified from the Alpe-DPD study participants to compare toxicity. To perform matching, we first studied associations of factors with toxicity one by one. Automatic matching (in a 1:3 ratio) was performed based on the three parameters that were most strongly associated with toxicity: treatment regimen, tumour type, and disease stage. If more than three eligible wild-type controls that fulfilled all matching criteria were available, these matches were selected at random from these eligible controls. Fisher’s exact test (conducted in SPSS) was conducted to compare the incidence of severe toxicity between deleterious variant carriers and their matched controls as the variant frequency was low. Due to the small sample size, we did not include a random effect for the matching cluster. Matching can improve the stability of statistical models as the matching variables do not have to be included in the model. For comparison, we have also analysed the non-matched sample with a logistic regression including the matching variables as covariates (conducted in R). Statistical analyses were conducted using SPSS version 25.0 and R version 4.2.3.

### Genome-wide association study

Genotyping was performed at the Human Genotyping Facility of the Erasmus University Medical Center, using the Infinium Global Screening Array (GSA) v1.0 [14]. The array contains 692,842 SNPs and includes rare variants with allele frequencies < 1%. A minor allele frequency (MAF) threshold of 0.5% was used for the primary analysis. 1000 Genomes reference phase 3 GRCh37.p13 was used to impute the data [15, 16] Quality control (QC) checks can be found in the Additional file 1: Text “*GWAS—Quality control*”. Genetic variants were tested for an association with the onset of severe fluoropyrimidine-induced toxicity. The primary outcome was severe (grade ≥ 3) fluoropyrimidine-induced toxicity, compared to grade ≤ 1 fluoropyrimidine-induced toxicity. Grade 2 toxicity was excluded from the primary analysis to maximize the contrast between toxicities (Fig. 1). Additionally, as a sensitivity analysis, severe fluoropyrimidine-induced toxicity was also compared to grade ≤ 2 fluoropyrimidine-induced toxicity. Gender, age, baseline body surface area (BSA), and treatment regimen (grouped as previously published [5]) were used as pre-specified covariates. Statistical analyses were performed in R statistics version 4.3.2 [17]. Base packages stats, survival, and MASS were used to evaluate logistic, Cox, and ordinal regression analyses, respectively. A *p* value threshold of ≤ 5 × 10^−8^ was used for determining significance at the genome-wide level. Post-association QC was performed by visual inspection of Quantile–Quantile (QQ) plots of *p* values of association tests and computation of the inflation factor. Online databases (Linkage-Disequilibrium tools, hapreg, and genome browser) were used to explore possible biological mechanisms of genome-wide associated or suggestive novel SNPs [18–20].

#### Power analysis

We performed power calculations based on the marginal event rate of 34% for the primary outcome. We based the calculations on 559 individuals (or 1118 alleles). Based on several allele frequencies, we calculated detectable effect size for a power of 80% and an alpha level of 5e − 8. For allele frequencies of 10%, 25%, and 50%, detectable ORs of risk alleles are 3.8, 2.4, and 2.2, respectively (Additional file 1: Table S2).

## Results

### Cohort

**Fig. 2:**
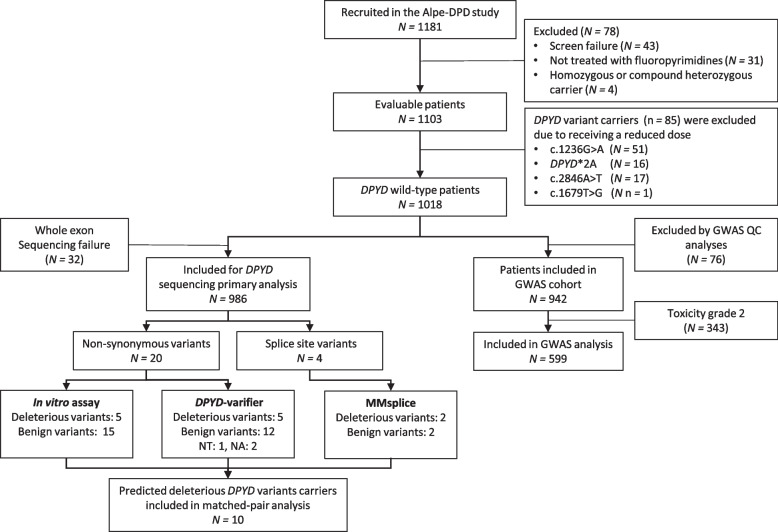
Flowchart of patient inclusion. Patients who experienced grade 2 toxicity were excluded from the GWAS analyses to maximize the contrast between severe and non-severe toxicity. Abbreviations: QC, quality control; DPYD, gene encoding dihydropyrimidine dehydrogenase; NT, not tested, NA, prediction not available

Patient characteristics are shown in Table 1. In total, 1181 were included in the Alpe-DPD study, of which 1103 were evaluable (Fig. 2). Of these, 85 *DPYD* variant carriers (DPYD*2A, c.1236G > A/HapB3, c.2846A > T, or c.1679 T > G) were treated with a reduced dose and consequently excluded from analyses, resulting in 1018 patients being evaluable for *DPYD* sequencing and GWAS analysis. Whole exon sequencing failures and GWAS quality control checks led to the exclusion of 32 and 74 patients, resulting in 986 and 942 patients being included in the *DPYD* sequencing analysis and GWAS, respectively. As stated in the methods section, patients with grade 2 toxicity were disregarded in the GWAS analysis, leading to 599 patients in the GWAS cohort.

### *DPYD* sequencing and variant function prediction

A total of 24 non-synonymous, frameshift, and splice site variants were detected in 986 individuals (excluding patients carrying any of the four known variants (DPYD*2A, c.1236G > A, c.2846A > T, and c.1679 T > G) tested for in the Alpe-DPD study. Of these variants, 20 were in exons and four were in introns (Table 2). The frequencies and results of the functional assessment with the in vitro assay and the MMsplice are described in Table 2. In total, seven rare deleterious variants were identified, which were carried by 10 individuals. Five variants in the coding region (c.1670C > T, c.1913 T > C, c.1925 T > C, c.506delC, and c.731A > C) and two variants in the flanking splice region (c.1740 + 1G > T and c.763 − 2A > G) were predicted to be deleterious. Of these seven variants, only c.1670C > T and c.763 − 2A > G have been reported previously. The remaining seventeen non-synonymous variants were predicted benign, of which 3 have not yet been previously noted in dbSNP. None of the 24 variants were classified as decreased function or no function allele according to the CPIC guideline.

**Table 2:** Frequencies and results of functional assessment of all variants

**Non-synonymous and frameshift variants**						
**Transcript change**	**dbSNP**	**Amino acid change**	**In vitro assay**	***DPYD*-varifier**	**Carriers, No. (%)**^**c**^	**Observed severe toxicity, No. (%)**^**d**^
c.1670C > T	rs754125729	T557I	Deleterious	Deleterious	2 (0.2%)	0 (0)
c.1913 T > C	NA	I638T	Deleterious	Deleterious	2 (0.2%)	0 (0)
c.1925 T > C	NA	M642Ts	Deleterious	Deleterious	2 (0.2%)	1 (50%)
c.506delC	NA	c.506delC	Deleterious^a^	NT^**b**^	1 (0.1%)	1 (100%)
c.731A > C	NA	E244A	Deleterious	Deleterious	1 (0.1%)	0 (0)
c.1218G > A	rs61622928	M406I	Neutral^a^	Neutral	5 (0.5%)	0 (0)
c.1601G > A	rs1801158	S534N	Neutral^a^	Neutral	46 (4.7%)	13 (28.3%)
c.1627A > G	rs1801159	I543V	Neutral^a^	Neutral	338 (34.3%)	81 (24.0%)
c.1796 T > C	rs147601618	M599T	Neutral^a^	Neutral	1 (0.1%)	0 (0)
c.2087G > A	rs778298325	R696H	Neutral	Neutral	2 (0.2%)	1 (50%)
c.2194G > A	rs1801160	V732I	Neutral^a^	Neutral	98 (9.9%)	22 (22.4%)
c.2194G > T	NA	V732F	Neutral	Deleterious	1 (0.1%)	1 (100%)
c.2621A > G	rs1164428597	K874R	Neutral	Neutral	1 (0.1%)	1 (100%)
c.2806G > T	rs137878450	G936C	Neutral^a^	Neutral	1 (0.1%)	1 (100%)
c.3067C > A	rs114096998	P1023T	Neutral^a^	NA	2 (0.2%)	2 (100%)
c.482A > G	NA	E161G	Neutral	Neutral	1 (0.1%)	0 (0)
c.496A > G	rs2297595	M166V	Neutral^a^	Neutral	166 (16.8%)	49 (29.5%)
c.768 T > G	rs556933127	I256M	Neutral	Neutral	3 (0.3%)	0 (0)
c.775A > G	rs45589337	K259E	Neutral^a^	Neutral	13 (1.3%)	2 (15.4%)
c.85 T > C	rs1801265	C29R	Neutral^a^	NA	362 (36.7%)	78 (21.5%)
**Splice site variants**						
**c**	**dbSNP**		**MMsplice**		**Carriers, No. (%)**^**c**^	**Observed severe toxicity, No. (%)**^**d**^
c.763 − 2A > G	rs1300669537		Deleterious		1 (0.1%)	0 (0)
c.1740 + 1G > T	NA		Deleterious		1 (0.1%)	1 (100%)
c.1905C > T^e^	rs3918289		Neutral		2 (0.2%)	0 (0)
c.1129 − 3delT	NA		Neutral		1 (0.1%)	0 (0)

^a^The in vitro assessment results of these variants have been published previously [10–112][10–112]. The primer sequences used to perform the novel site-directed mutagenesis on the expression plasmids are included in the Additional file 1: Table S1

^**b**^Outside of structurally defined regions of human DPD protein and therefore cannot be classified using *DPYD-*Varifier*. NT*, not testable; *NA*, not assigned

^c^The frequency of variant carriers was calculated in successfully sequenced patients (*n* = 986)

^d^The percentage of observed severe toxicity was calculated based on the corresponding number of variant carriers

^e^c.1905C > T is near exon/intron boundaries that could be tested by MMSplice and it cannot be tested by the in vitro assay as it does not directly change the amino acid

Out of the patients who carried predicted deleterious variants, 3 of 10 (30%) patients developed severe toxicity. None of the 10 deleterious variants was related to a statistically significant increased risk of several toxicities compared with matched controls who did not carry any deleterious DPYD variant, of which, 16.7% (5 out of 30 patients) experienced severe toxicities (OR 2.143, *P* < 0.388; Table 3). In addition, all grade toxicity was comparable between carriers and matched non-carriers (OR 2.25, *p* < 0.656; Table 3). The patient characteristics of ten carriers and their matched control are shown in the Additional file 2: Table S3. In a post hoc exploratory analysis also no statistically significant difference in severe toxicity was found between patients carrying a predicted deleterious variant (*n* = 10) and 976 non-carriers (logistic regression: OR 1.49 CI 0.38 to 5.87, *P* = 0.57, Additional file 2: Table S4).

**Table 3:** Matched pair analysis of novel deleterious variants

	Predicted deleterious variants carriers (*N* = 10)	Matched patients without deleterious variants (*N* = 30)	*P* value	Odds ratio (95% CI)	Positive predictive value	Negative predictive value
≥ grade 1 toxicity	9 (90%)	24 (80%)	0.656	2.250 (0.237–21.367)	27.2	85.7
Severe toxicity (grade ≥ 3)	3 (30%)	5 (16.7%)	0.388	2.143 (0.408–11.255)	37.5	78.1

*Abbreviations*: *CI*, confidence interval

### Genome-wide association analysis

GWAS was assessed for severe (grade ≥ 3) toxicity and was compared to grade 0 or 1 toxicity in 599 patients (excluding 343 patients with grade 2 toxicity, Fig. 2). The number of patients varied per SNP due to genotype missingness, which was limited to up to 3% as per QC. An association test for severe fluoropyrimidine-induced toxicity (CTC-AE grades 3–5) was performed for a total of 4,650,899 markers. Gender, age, baseline BSA, and treatment type were included as covariates. The corresponding Manhattan and QQ plots are shown in the Additional file 2: Fig. S1 and Fig. S2. The inflation factor is 1.04. While none of the individual SNPs achieved genome-wide significance as per the pre-specified definition (*p* ≤ 5 × 10^−8^), five variants (rs17114875, rs367239, rs77579689, rs114105116, and rs12622722) showed a suggestive association with severe toxicity, with *p* values between 5 × 10^−8^ and 5 × 10^−6^. The closest annotated genes to rs171114875 are *PRKD1* and *MIR548AI.* The closest annotated gene to rs77579689 is *KHDRBS3*. The closest annotated genes to rs367239 are *VENTXP7* and *ZNF385D*. Additionally, rs367239 is in linkage disequilibrium with rs1396004 and rs341838 which are both SNPs located in *VENTXP7*. The other two suggestive variants are listed as intronic variants of the non-coding RNA gene *LOC101927414* (rs114105116) and protein-coding gene *COL6A3* (rs12622722). The 30 most significantly associated markers are shown in Table 4. None of these SNPs have previously been reported in publications or the ClinVar database of the National Center for Biotechnology Information (NCBI) [15].

**Table 4:** Thirty genetic variants with the lowest *p* values

Nr	Marker	Chr	Position	A0	A1	AF	*β*	*P* value
1	rs17114875*	14	29999987	G	A	0.409	− 0.65087	5.14 × 10^−07^
2	rs114105116*	4	138539880	T	A	0.020	3.124216	1.13 × 10^−06^
3	rs367239*	3	21421935	T	C	0.546	− 0.59915	2.45 × 10^−06^
4	rs12622722*	2	238269120	G	A	0.484	0.627528	4.55 × 10^−06^
5	rs77579689*	8	137130325	G	A	0.021	− 3.90131	4.64 × 10^−06^
6	rs74910762	8	81109425	C	A	0.044	1.419904	5.29 × 10^−06^
7	chr16:78157332:I	16	78157332	G	GTT	0.065	1.20317	5.55 × 10^−06^
8	rs12414693	10	97228795	C	T	0.259	0.672817	5.73 × 10^−06^
9	rs449973	3	21425977	C	G	0.548	− 0.56792	6.23 × 10^−06^
10	rs495426	12	31021833	A	G	0.689	0.661952	6.63 × 10^−06^
11	chr4:164083322:D	4	164083322	TG	T	0.051	1.624069	7.09 × 10^−06^
12	rs1722291	7	56238936	G	A	0.198	− 0.75534	7.81 × 10^−06^
13	rs147501714	15	102309786	G	A	0.041	2.183951	8.07 × 10^−06^
14	rs76146060	8	81120217	A	T	0.044	1.404553	8.12 × 10^−06^
15	rs12415681	10	97233085	T	C	0.258	0.657462	8.66 × 10^−06^
16	rs11595114	10	97231520	G	T	0.258	0.658815	8.67 × 10^−06^
17	rs12415079	10	97229543	G	C	0.257	0.660208	8.70 × 10^−06^
18	rs2344989	17	70924851	T	C	0.040	− 1.72137	8.96 × 10^−06^
19	rs8076418	17	70921917	T	C	0.042	− 1.70739	9.53 × 10^−06^
20	rs184137490	4	64175576	A	T	0.028	− 2.76836	9.54 × 10^−06^
21	rs2085003	2	5323126	A	T	0.930	1.524253	9.99 × 10^−06^
22	rs10742634	11	42623993	G	A	0.458	0.550361	1.01 × 10^−05^
23	rs77635577	16	61042468	A	T	0.108	− 0.96048	1.05 × 10^−05^
24	rs8067883	17	70921731	C	T	0.042	− 1.70883	1.06 × 10^−05^
25	rs4304264	7	89699913	A	T	0.191	− 0.76196	1.06 × 10^−05^
26	rs6501582	17	70921801	T	C	0.042	− 1.70574	1.06 × 10^−05^
27	rs8070810	17	70921851	G	A	0.042	− 1.70479	1.06 × 10^−05^
28	rs10895872	11	105604719	A	G	0.553	− 0.58102	1.06 × 10^−05^
29	rs9911437	17	70922305	T	C	0.042	− 1.70383	1.07 × 10^−05^
30	chr17:70923098:D	17	70923098	AC	A	0.042	− 1.7027	1.09 × 10^−05^

*Abbreviations*: *Nr*, number; *Chr*, chromosome; *A0*, nucleotide on allele 0; *A1*, nucleotide on allele 1; *AF*, allele frequency

Variants are selected on allele frequency > 0.01, *β* within − 5 to 5, and are separated from another variant with more than 10 bps. Variants suggestive of the onset of severe toxicity are marked with an asterisk (*)

## Discussion

While applying prospective *DPYD* genotyping to clinical practice has successfully reduced the incidence of severe toxicity, a substantial number of patients treated with fluoropyrimidines still experience severe treatment-related toxicity [5]. We hypothesized that in addition to the four established *DPYD* variants, other genetic variations in- and outside *DPYD* might be associated with the onset of severe fluoropyrimidine-related toxicity. Therefore, we performed comprehensive genetic analyses including whole exon sequencing of *DPYD* and a GWAS analysis in a large well-characterized cohort derived from a prospective clinical study consisting of 1103 mostly Caucasian patients (95%) treated with fluoropyrimidine-based chemotherapy [5]. Within *DPYD*, we detected 24 non-synonymous and splice site variants, of which 7 allele variants that were carried in 10 patients were predicted to be deleterious. In the matched-pair analysis, the carriers of these deleterious variants showed a statistically non-significant twofold higher risk of severe toxicity. These findings imply that patients with rare deleterious variants may be at increased risk of severe fluoropyrimidine-related toxicity.

Out of the 24 detected variants, 5 deleterious variants are novel and would have been missed with a pre-designed panel test, highlighting the potential of the combination of next-generation sequencing (NGS) with available functionality assessment tools in detecting deleterious variants and preventing life-threatening toxicity. Yet, despite analysis of a large cohort of over 1000 patients, the number of novel deleterious *DPYD* variants remains low. Moreover, it is challenging to connect these unique variants to clinical decisions or upfront dose reductions because of the risk of undertreatment, limiting clinical application. By contrast, a study consisting of 120 patients developing grade 3–5 fluoropyrimidine-related toxicity and 104 matched controls identified a significant increased risk of patients carrying at least one rare missense DPYD variant [21]. Therefore, additional studies on implementing these approaches are needed, especially in understudied populations, which are more likely to carry other deleterious *DPYD* variants in addition to the four commonly tested ones [9]. However, in our cohort, even after accounting for the additional deleterious variants in *DPYD*, unexplained severe fluoropyrimidine-induced toxicity remained. Potentially, this remaining toxicity is the result of genetic variation outside *DPYD.* Several GWAS studies have been performed in patients and cell lines in attempts to identify novel risk variants [22–24]. These previous studies failed to identify associations that reached genome-wide significance, possibly due to limitations including small sample size and focus on specific toxicities such as neutropenia or leucopenia [22–24]. Similarly, no variants in our GWAS reached genome-wide significance despite the comparatively large sample size and broader definition of fluoropyrimidine-associated toxicity, suggesting that non-genetic variables and/or more complex interactions between genetic components, with each exerting a small effect size, contribute to the occurrence of severe fluoropyrimidine induced toxicity. Polygenic risk models are an attractive approach to address this issue; however, such analyses require far more patients than are available in our study.

Although no genome-wide significant SNPs were identified, we did identify five variants suggestive of association with severe fluoropyrimidine-induced toxicity that might provide insight into possible alternative mechanisms that contribute to fluoropyrimidine toxicity. To the best of our knowledge, these SNPs have not been previously described in relation to fluoropyrimidines [25, 26].

One trade-off of not considering patients who experienced grade 2 toxicity is that not all patients of the cohort are included in the association analysis. Therefore, we conducted a sensitivity analysis by including the patients with grade 2 fluoropyrimidine-related toxicity (grade 0–2 vs. grade 3–5), thereby increasing the number of patients while reducing the contrast between toxicities. Yet, this did not result in a different outcome (Additional file 2: Fig. S3 and Table S5). Furthermore, as toxicities can differ between capecitabine and 5-FU, we repeated the GWAS with patients receiving capecitabine as this was the majority of patients (494 (82%) of 599). This analysis did not result in a different outcome.

Our results indicate that *DPYD* exonic variants, especially predicted deleterious variants, as well as the five GWAS variants that were found to be suggestive of association with severe fluoropyrimidine-induced toxicity, are candidate SNPs that are valuable for further study. However, a substantial part of the observed fluoropyrimidine-related toxicity remains unexplained and other explanations such as the contribution of variants in non-coding regions and rare variants outside *DPYD* should also be considered. Recently, the association between rare variants in *DPYS*, a gene involved in the catabolic pathway with *DPYD*, and fluoropyrimidine-related severe toxicity was reported, which suggested a fourfold increased risk of cumulative severe toxicity [25]. Furthermore, while exon sequencing had a limited contribution to explaining the remaining severe fluoropyrimidine-toxicity in our patient population consisting of mostly Caucasians (95%), other genetic variants may be present in more ethnically diverse study populations as it is well-known that these facilitate the identification of genetic risk factors [26]. Additional studies in populations with greater ancestral diversity are therefore needed. Our analyses applied a toxicity definition of overall ≥ grade 3 toxicity during the entire treatment duration as used previously [25]. However, the effect of genetic contributors to toxicity might be more obvious in the earlier cycles of treatment. To further address this, we performed a sensitivity analysis with overall ≥ grade 3 toxicity during the first two treatment cycles as the endpoint for both the GWAS and the matched pair analyses. These analyses did not detect any new signals (Additional file 2: Fig. S4 and Table S6, S7, S8).

In conclusion, our results from *DPYD* exon sequencing and GWAS analysis suggest that at a population level it is not likely that, besides the four established *DPYD* variants, genetic variants either inside or outside *DPYD* have a clinically relevant contribution to severe fluoropyrimidine-induced toxicity in patients treated with fluoropyrimidines. However, at patient level, it cannot be excluded—based upon this study—that a rare variant is causing toxicity. Therefore, larger studies, in more diverse populations are needed to identify these additional variants.

## Conclusions

Results from *DPYD* exon sequencing and GWAS analysis did not identify additional genetic variants associated with severe fluoropyrimidine-related toxicity, which suggests that testing for single markers at a population level currently has limited clinical value. Identifying additional variants on an individual level is still promising to explain fluoropyrimidine-related severe toxicity. In addition, studies with larger sample sizes, in more diverse cohorts are needed to identify potential clinically relevant genetic variants related to severe fluoropyrimidine toxicity.

### Supplementary Information

Additional file 1. Supplementary methods. Supplementary information regarding the inclusion and exclusion criteria of the Alpe-DPD study and the quality control of the GWAS. Table S1: In vitro assay - The primer sequences used to perform site-directed mutagenesis on the expression plasmids. Table S2. The power analysis of the GWAS.

Additional file 2. Supplementary results. Table S3. The characteristics of patients included in the matched-pair analysis. Table S4. The severe toxicity between the predicted deleterious variants carriers and non-carriers compared by the logistic regression. Fig S1: Manhattan plot for association with severe fluoropyrimidine-induced toxicity. Fig S2: QQ-plot of p-values. Fig S3: Manhattan plot for association with severe fluoropyrimidine-induced toxicity (Sensitivity analysis). Table S5: Thirty genetic variants with the lowest p values (Sensitivity analysis). Table S6. Severe toxicities in the first two cycles and the entire treatment duration. Table S7. The association between novel DPYD deleterious variants and ≥ grade 3 toxicity in the early two cycles of treatment. Fig S4: Manhattan plot for association with severe fluoropyrimidine-induced toxicity (Sensitivity analysis). Table S8: Table of the top 30 associated variants related to severe toxicity in the early two cycles.

## Data Availability

Sequence and GWAS data that support the findings of this study are available in our European Genome and Phenotype Archive (EGA) account EGA-box-1314 accession code EGAS00001007855.
