# Supplementary material for: Discovering novel germline genetic variants linked to severe fluoropyrimidine-related toxicity in- and outside DPYD
Source: Genome Med. 2024 Aug 15;16:101. doi: 10.1186/s13073-024-01354-z (PMC11325793; doi:10.1186/s13073-024-01354-z)
Supplement: Supplementary file 1 — Additional file 1. Supplementary methods. Supplementary information regarding the inclusion and exclusion criteria of the Alpe-DPD study and the quality control of the GWAS. Table S1: In vitro assay - The primer sequences used to perform site-directed mutagenesis on the expression plasmids. Table S2. The power analysis of the GWAS. [file 13073_2024_1354_MOESM1_ESM.docx]

**Additional file 1: Supplementary methods**

**Inclusion criteria of the Alpe-DPD study**

Inclusion criteria for all participating patients:

1. Pathologically confirmed malignancy for which treatment with a fluoropyrimidine is
2. considered to be in the patient’s best interest
3. Age ≥ 18 years
4. Able and willing to give written informed consent
5. WHO performance status of 0, 1 or 2
6. Life expectancy of at least 12 weeks
7. Able to swallow and retain oral medication
8. Able and willing to undergo blood sampling for pharmacogenetic and phenotyping analysis
9. Minimal acceptable safety laboratory values
   1. ANC of ≥ 1.5 x 109 /L
   2. Platelet count of ≥ 100 x 109 /L
   3. Hepatic function as defined by serum bilirubin ≤ 1.5 x ULN, ALAT and ASAT ≤ 2.5 x ULN; in case of liver metastases ALAT and ASAT ≤ 5 x ULN.
   4. Renal function as defined by serum creatinine ≤ 1.5 x ULN or creatinine clearance ≥ 60 ml/min (by Cockcroft-Gault formula).

Additional inclusion criteria for patients in subgroups of the study:

1. Able and willing to undergo blood sampling and breath sampling at several time points
2. Able and willing to receive uracil for the test dose assay
3. Able and willing to receive [2-^13^C]-labeled uracil for the breath test

**Exclusion criteria of the Alpe-DPD study**

1. Prior treatment with fluoropyrimidines
2. Patients with known substance abuse, psychotic disorders, and/or other diseases
3. expected to interfere with study or the patient’s safety
4. Women who are pregnant or breast feeding
5. Both men and women who refuse to use reliable contraceptive methods throughout the
6. study (adequate contraceptive methods are: condom, sterilization, other barrier
7. contraceptive measures preferably in combination with condoms)
8. Patients with a homozygous polymorphic genotype or compound heterozygous
9. genotype for DPYD.

**Table S1:** *In vitro* assay - The primer sequences used to perform site-directed mutagenesis on the expression plasmids

| Variants | Primer |
| --- | --- |
| T557I | 5’ TATCAATGATTCGAAGAGCTTTTGAAGCTGG  5’ TGCTGGTGGCTGGAGTTGC |
| I638T | 5’ CTGCTAGCATTATGTGCAGTTACAATAAAAATGAC  5’ TCACAATGTTGTCTGGAAAGTCAGCC |
| M642T | 5’ CGTGCAGTTACAATAAAAATGACTGGACGG  5’ TAATGCTAGCAATCACAATGTTGTCTGGA |
| R696H | 5’ ACTGGGTTAGGCAAGCTGTTCAG  5’ GGCAGATGTTCCGCACCAGC |
| V732F | 5’ TTTACAGCCACCAACACTGTCTCAG  5’ GCCATTGGCACCACCTTCCT |
| K874R | 5’ GGAAACTGCCAAGTTTTGGACCTTATCT  5’ TGTCCATGAGTTCAGCTATACGTGG |
| E161G | 5’ GGGTATTCAAAGCAATGAGTATCCCACA  5’ CAGTAGCAAATTGCTGCAATCCACC |
| E244A | 5’ CGATTGAGCTAATGAAGGACCTTGGT  5’ CAAAATTCACTACATCATACGGCAGCC |
| I256M | 5’ GTGCGGTAAAAGCCTTTCAGTGAATG  5’ ATTATCTTTACACCAAGGTCCTTCATTAGCTC |

***GWAS - Quality control***

Quality control (QC) checks were performed using software R version 3.5.0^1^ and PLINK software, version 1.07.^2, 3^ Patients were excluded from analyses based on an individual genotype call rate <97%, gender mismatch between reported and estimated sex based on genotypes of the X-chromosome (using PLINK), or excess of heterozygous genotypes as measured by the inbreeding coefficient. Patients were removed from the analysis if the inbreeding statistic F>0∙1, which was judged to be outlying. Genetic markers were excluded based on a SNP call rate <97% and a p-value ≤10^-7^ for the Hardy-Weinberg equilibrium (HWE) goodness-of-fit test. After exclusion of patients and markers in these marginal QCs, the remaining set was used for integrative QC assessment. To evaluate the possibility of population stratification or outliers, multidimensional scaling (MDS) analysis was performed in PLINK. In addition, pairwise identity by state (IBS) / identity by descent (IBD) statistics were calculated to assess duplicates. MDS, IBS, and IBD were computed using PLINK. Patients who were identified as outliers based on IBS clustering were excluded from the analysis. MDS coordinates were extracted and used as covariates in the association analysis. SNP imputation was performed using the programs *shapeit* and *impute2*^4, 5^ with default parameters in which the reference panel 1000Genomes build version 3 was used with a total, ‘cosmopolitan’, set of individuals.^6^ An MDS plot was created to compare the self-reported ethnicity of patients.

***Power analysis***

**Table S2.** The power analysis of the GWAS. A logistic regression of a binary response variable (Y) on a binary independent variable (X) with a sample size of 1118 observations (of which 95% are in the group X=0 and 5% are in the group X=1) achieves 80% power at a 5x10^-8^ significance level to detect a change in Prob(Y=1) from the baseline value of 0,340 to NA. This change corresponds to an odds ratio of NA. A two-sided Wald test is used.

| Power | N | Percent X=1 | P0 | P1 | ORyx | Alpha | Beta |
| --- | --- | --- | --- | --- | --- | --- | --- |
| 0,8000 | 1118 | 10% | 0,340 | 0,659 | 3,758 | 5x10^-8^ | 0,2000 |
| 0,8000 | 1118 | 25% | 0,340 | 0,555 | 2,424 | 5x10^-8^ | 0,2000 |
| 0,8000 | 1118 | 50% | 0,340 | 0,528 | 2.170 | 5x10^-8^ | 0,2000 |

*Logistic regression equation: Log(P/(1-P)) = β0 + β1×X, where P = Pr(Y = 1|X) and X is binary.*

*Power is the probability of rejecting a false null hypothesis. N is the sample size. Percent X=1 is the percent of the sample in which the exposure is 1 (present). P0 is the response probability at X = 0. That is, P0 = Pr(Y = 1|X = 0). P1 is the response probability at X = 1. That is, P1 = Pr(Y = 1|X = 1).*

*ORyx is the odds ratio under the alternative hypothesis. That is, it is [P1/(1-P1)]/[P0/(1-P0)]. Alpha is the probability of rejecting a true null hypothesis. Beta is the probability of accepting a false null hypothesis. NA not assigned.*

**References:**

**1**. Team. RCore: R: A Language and Evironment for Statistical Computinghttps://www.R-project.org., 2018

**2**. Purcell S: PLINK 1.07. http://pngu.mgh.harvard.edu/purcell/plink/http://pngu.mgh.harvard.edu/purcell/plink/, 2018

**3**. Purcell S, Neale B, Todd-Brown K, et al: PLINK: A tool set for whole-genome association and population-based linkage analyses. American Journal of Human Genetics 81:559–575, 2007

**4**. Delaneau O, Marchini J, Zagury JF: A linear complexity phasing method for thousands of genomes. Nature Methods 9:179–181, 2012

**5**. Howie BN, Donnelly P, Marchini J: A flexible and accurate genotype imputation method for the next generation of genome-wide association studies. PLoS Genetics 5, 2009

**6**. Marchini J, Howie B, Myers S, et al: A new multipoint method for genome-wide association studies by imputation of genotypes. Nature Genetics 39:906–913, 2007
