# Supplementary material for: Discovering novel germline genetic variants linked to severe fluoropyrimidine-related toxicity in- and outside DPYD
Source: Genome Med. 2024 Aug 15;16:101. doi: 10.1186/s13073-024-01354-z (PMC11325793; doi:10.1186/s13073-024-01354-z)
Supplement: Supplementary file 2 — Additional file 2. Supplementary results. Table S3. The characteristics of patients included in the matched-pair analysis. Table S4. The severe toxicity between the predicted deleterious variants carriers and non-carriers compared by the logistic regression. Fig S1: Manhattan plot for association with severe fluoropyrimidine-induced toxicity. Fig S2: QQ-plot of p-values. Fig S3: Manhattan plot for association with severe fluoropyrimidine-induced toxicity (Sensitivity analysis). Table S5: Thirty genetic variants with the lowest p values (Sensitivity analysis). Table S6. Severe toxicities in the first two cycles and the entire treatment duration. Table S7. The association between novel DPYD deleterious variants and ≥ grade 3 toxicity in the early two cycles of treatment. Fig S4: Manhattan plot for association with severe fluoropyrimidine-induced toxicity (Sensitivity analysis). Table S8: Table of the top 30 associated variants related to severe toxicity in the early two cycles. [file 13073_2024_1354_MOESM2_ESM.docx]

**Additional file 2: Supplementary results**

*DPYD* sequencing and variant function prediction

**Table S3.** The characteristics of patients included in the matched-pair analysis

|  | Predicted deleterious variants carriers  (n=10) | Matched patients without deleterious variants  (n=30) |  |
| --- | --- | --- | --- |
| Gender  *Male*  *Female* | 7 (70%)  3 (30%) | 15 (50%)  15 (50%) |  |
| Age in years (median, IQR) | 63 (55─69) | 63 (55─70) |  |
| Ancestry  *White* | 10 (100%) | 30 (100%) |  |
| BSA (media, IQR) | 1.9 (1.8─2.1) | 2.0 (1.8─2.1) |  |
| WHO performance status  *0*  *1*  *2*  *Not specified* | 6 (60%)  4 (40%)  0  0 | 11 (37%)  15 (50%)  2 (7%)  2 (7%) |  |
| Number of treatment cycles | 3 (2-6) | 3 (2-6) |  |

Data in n (%) or median (IQR). Abbreviations: BSA-Body surface area. WHO-World Health Organization.

**Table S4.** The severe toxicity between the predicted deleterious variants carriers and non-carriers comparied by the logistic regression.

|  | Estimate | Std.Error | z value | Pr(>\|z\|) | OR | 95% Confidence intervel |
| --- | --- | --- | --- | --- | --- | --- |
| Intercept | -1.54 | 0.15 | -10.35 | <2e-16 | 0.22 | 0.16-0.29 |
| Predicted deleterious variants | 0.40 | 0.70 | 0.57 | 0.57 | 1.49 | 0.38-5.87 |
| Treatment strategy | 0.09 | 0.04 | 2.55 | 0.01 | 1.10 | 1.02-1.18 |
| Tumor type | -0.00 | 0.00 | -0.64 | 0.53 | 0.10 | 0.99-1.00 |
| Disease stage | 0.01 | 0.01 | 0.83 | 0.41 | 1.01 | 0.99-1.03 |

*Genome-wide association analysis*

*Genotyping and quality control*

A set of 692,367 markers was genotyped. After several QC steps, 186,920 markers were excluded. Of these, 18,114 markers (2∙6%) were excluded based on a deviation from Hardy-Weinberg equilibrium (HWE). Filtering for allele frequencies (threshold 0∙5%) resulted in the exclusion of 147,607 markers (21∙3%). In total, 23,835 markers (3∙4%) were excluded based on the missing data analysis (missingness cut-off at 10%). Of the abovementioned excluded markers, 2,636 had multiple QC failures. In total, 505,447 markers met the QC for statistical analyses. After imputation with using the 1000 Genomes dataset as a reference panel 4,650,899 variants were available for statistical analyses. In the integrative QC, individuals and markers from the marginal QC steps were excluded. An MDS analysis was executed to detect population stratification. IBD/IBS clustering was executed to assess duplicates. No individuals were excluded based on this analysis.

**Fig S1:**

**
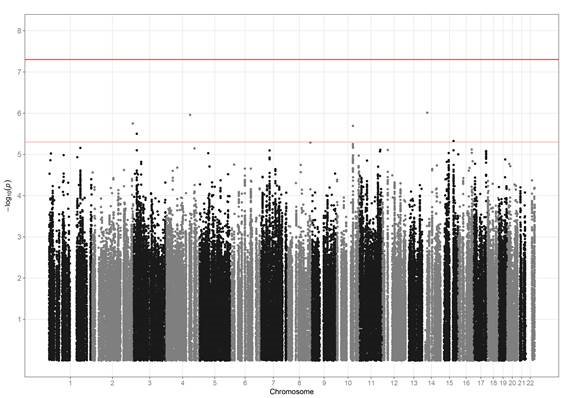
**

**Fig S1:** Manhattan plot for association with severe fluoropyrimidine-induced toxicity. Manhattan plot for association with severe fluoropyrimidine-induced toxicity (grade ≥3), including de covariates gender, age, baseline BSA, and treatment type. Genome-wide significance of the association with the onset of severe fluoropyrimidine-induced toxicity is indicated by the upper dark red line (p value of ≤5x10-8). Suggestive association is indicated by the lower red line (p value of ≤5x10-6). No SNPs were found to be significantly associated with severe fluoropyrimidine-induced toxicity. Five SNPs were found to be suggestive for association with severe fluoropyrimidine-induced toxicity, shown in Table 4. Abbreviations: BSA: body surface area; SNPs: single nucleotide polymorphisms.

**Fig S2:**

**
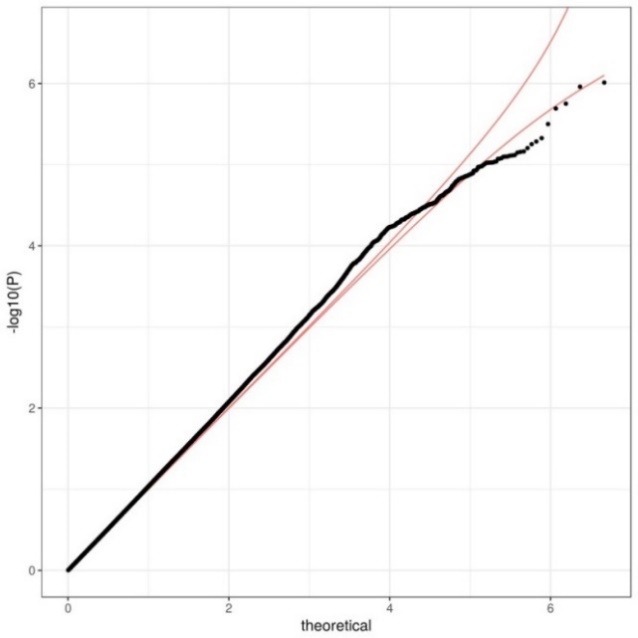
**

**Fig S2:** QQ-plot of p-values. The Quantile-Quantile (QQ)-plot shows the extent to which the observed distribution of the test statistic follows the theoretical null distribution. The inflation factor was λ=1∙04.

**Fig S3:**


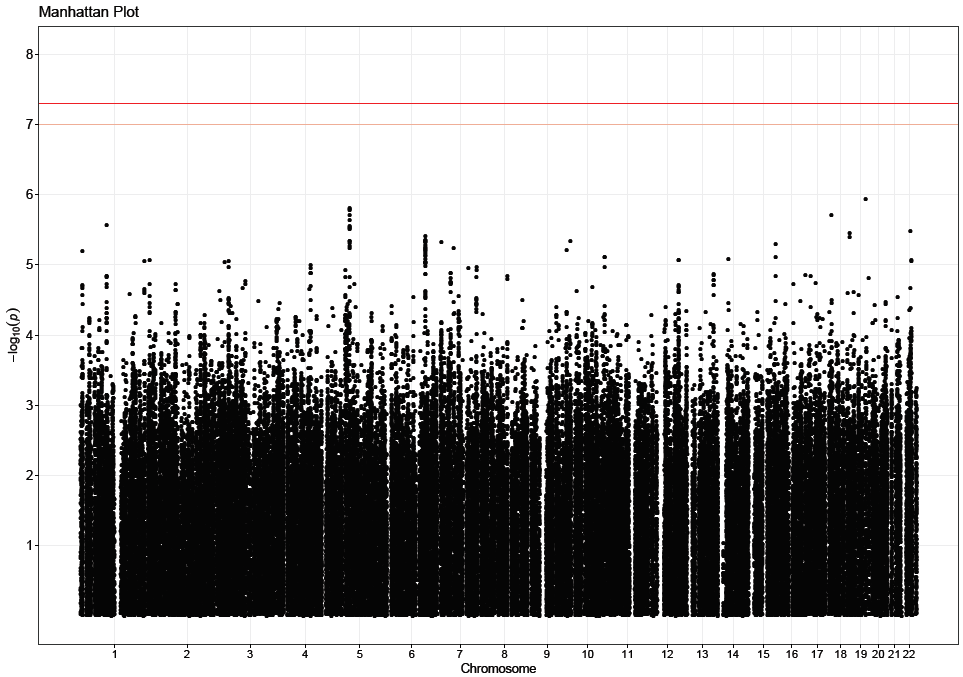


**Fig S3:** Manhattan plot for association with severe fluoropyrimidine-induced toxicity. Manhattan plot for association with fluoropyrimidine-induced toxicity (grade ≥2), including de covariates gender, age, baseline BSA, and treatment type. Abbreviations: BSA: body surface area; SNPs: single nucleotide polymorphisms.

**Table S5:** Thirty genetic variants with the lowest p values
Variants are selected on allele frequency >0∙01, β within -5 to 5, and are separated from another variant with more than 10 bps. Abbreviations: Nr: number; Chr: chromosome; A0: nucleotide on allele 0; A1: nucleotide on allele 1; AF: allele frequency.

| **Nr.** | **Marker** | **Chr** | **Position** | **A0** | **A1** | **AF** | **β** | ***P* value** |
| --- | --- | --- | --- | --- | --- | --- | --- | --- |
| 1 | rs2627645 | 19 | 45705323 | C | A | 0∙932 | -1∙61014 | 1∙18 x 10^-06^ |
| 2 | rs2897989 | 5 | 56313932 | A | C | 0∙248 | -1∙17743 | 1∙59 x 10^-06^ |
| 3 | rs115429202 | 5 | 56312774 | T | G | 0∙247 | -1∙18033 | 1∙60 x 10^-06^ |
| 4 | rs57877390 | 5 | 56312654 | T | A | 0∙247 | -1∙17787 | 1∙65 x 10^-06^ |
| 5 | rs518004 | 18 | 7485914 | C | A | 0∙139 | -1∙65712 | 1∙98 x 10^-06^ |
| 6 | rs62356628 | 5 | 56315591 | A | G | 0∙248 | -1∙17094 | 1∙98 x 10^-06^ |
| 7 | rs62356625 | 5 | 56314320 | T | C | 0∙254 | -1∙14236 | 2∙35 x 10^-06^ |
| 8 | rs75025959 | 1 | 96902149 | C | T | 0∙828 | -1∙06894 | 2∙77 x 10^-06^ |
| 9 | rs62356624 | 5 | 56314210 | A | G | 0∙251 | -1∙13938 | 2∙83 x 10^-06^ |
| 10 | rs62356627 | 5 | 56315157 | C | T | 0∙251 | -1∙13858 | 2∙95 x 10^-06^ |
| 11 | rs62356626 | 5 | 56314910 | G | T | 0∙251 | -1∙13827 | 3∙01 x 10^-06^ |
| 12 | rs17732457 | 5 | 56301089 | T | C | 0∙262 | -1∙09054 | 3∙08 x 10^-06^ |
| 13 | rs72396379 | 22 | 28868536 | GTGTA | G | 0∙413 | 0∙831405 | 3∙38 x 10^-06^ |
| 14 | chr18:68930999:D | 18 | 68930999 | CTAT | C | 0∙026 | 2∙225657 | 3∙59 x 10^-06^ |
| 15 | rs142815469 | 6 | 130656827 | A | G | 0∙026 | 1∙835159 | 3∙96 x 10^-06^ |
| 16 | rs117010467 | 18 | 68949442 | A | G | 0∙03 | 2∙161431 | 4∙12 x 10^-06^ |
| 17 | rs74537901 | 6 | 130646607 | G | A | 0∙027 | 1∙767477 | 4∙49 x 10^-06^ |
| 18 | rs117198473 | 6 | 130651408 | G | A | 0∙027 | 1∙767392 | 4∙50 x 10^-06^ |
| 19 | chr6:130656050:D | 6 | 130656050 | CCAGCAGA | C | 0∙027 | 1∙767073 | 4∙52 x 10^-06^ |
| 20 | rs75556955 | 6 | 130643789 | C | A | 0∙027 | 1∙767295 | 4∙53 x 10^-06^ |
| 21 | rs117001051 | 6 | 130660192 | G | A | 0∙027 | 1∙766165 | 4∙57 x 10^-06^ |
| 22 | chr5:56308030:D | 5 | 56308030 | CAT | C | 0∙275 | -1∙04733 | 4∙65 x 10^-06^ |
| 23 | rs12188896 | 5 | 56298297 | G | A | 0∙259 | -1∙07756 | 4∙68 x 10^-06^ |
| 24 | rs145061688 | 10 | 8347578 | A | G | 0∙031 | -151∙26 | 4∙68 x 10^-06^ |
| 25 | rs117376718 | 7 | 13342578 | C | T | 0∙027 | 1∙889196 | 4∙70 x 10^-06^ |
| 26 | rs75361266 | 6 | 130664619 | C | T | 0∙027 | 1∙763235 | 4∙73 x 10^-06^ |
| 27 | rs116940099 | 6 | 130665596 | G | A | 0∙027 | 1∙761975 | 4∙80 x 10^-06^ |
| 28 | chr5:56297015:D | 5 | 56297015 | AG | A | 0∙259 | -1∙07594 | 4∙92 x 10^-06^ |
| 29 | rs61201669 | 5 | 56295995 | T | C | 0∙259 | -1∙07489 | 5∙07 x 10^-06^ |
| 30 | rs117481946 | 6 | 130668660 | G | C | 0∙027 | 1∙755796 | 5∙12 x 10^-06^ |

*Results of GWAS and matched-pair analysis with the endpoint of fluoropyrimidine-related severe toxicity in the early two cycles*

**Table S6**. Severe toxicities in the first two cycles and the entire treatment duration.

|  | Number of grade ≥ 3 toxicity in the first two cycles | Number of grade ≥ 3 toxicity in the entire treatment duration |
| --- | --- | --- |
| All patients (n = 1103) | 179 (16.2%) | 264 (23.9%) |
| *2A carriers (n = 16) | 4 (25%) | 5 (31.3%) |
| Novel variants carriers (n = 10) | 3 (33.3%) | 3 (33.3%) |
| Matched controls  ( n =30) | 4 (13.3%) | 5 (16.7%) |

**Table S7**. The association between novel *DPYD* deleterious variants and ≥ grade 3 toxicity in the early two cycles of treatment.

|  | Predicted deleterious variants carriers  (*N*=10) | Matched patients without deleterious variants (*N*=30) | *P* value | Odds ratio  (95% CI) | Positive predictive value | Negative predictive value |
| --- | --- | --- | --- | --- | --- | --- |
| Severe toxicity (grade≥ 3) | 3 (30%) | 4 (13.3%) | 0.338 | 2.786  (0.502-15.462) | 42.9 | 78.8 |

| 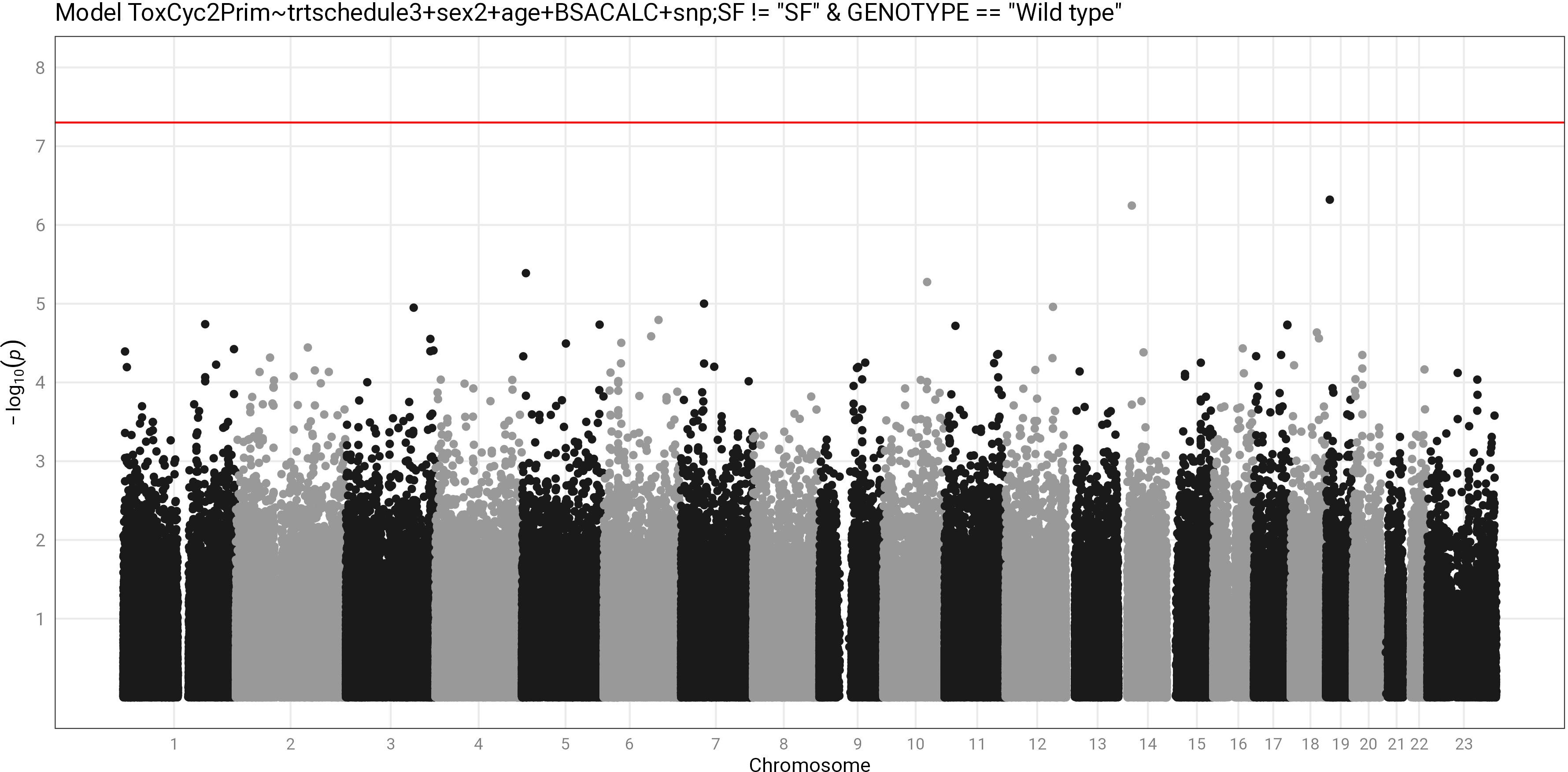 | | | | | | | | |
| --- | --- | --- | --- | --- | --- | --- | --- | --- |
| **Fig S4**: Manhattan plot for association with severe fluoropyrimidine-induced toxicity. Manhattan plot for association with fluoropyrimidine-induced toxicity (grade ≥2), including de covariates gender, age, baseline BSA, and treatment type. Abbreviations: BSA: body surface area; SNPs: single nucleotide polymorphisms.    **Table S8:** Table of the top 30 associated variants related to severe toxicity in the early two cycles. Abbreviations: Nr: number; Chr: chromosome; A0: nucleotide on allele 0; A1: nucleotide on allele 1; AF: allele frequency. | | | | | | | | |
| **Nr.** | **Marker** | **Chr** | **Position** | **A0** | **A1** | **AF** | **β** | ***P* value** |
| 1 | rs383273 | 19 | 8317961 | C | A | 0.04 | -16.38 | 4.78x10^-07^ |
| 2 | rs17114875 | 14 | 29999987 | A | A | 0.41 | -0.7 | 5.67x10^-07^ |
| 3 | rs74482613 | 5 | 8927592 | T | C | 0.06 | 1.15 | 4.09x10^-06^ |
| 4 | rs114494406 | 10 | 97096357 | T | T | 0.02 | 2.01 | 5.30x10^-06^ |
| 5 | rs73349015 | 7 | 50762833 | C | A | 0.05 | -1.97 | 9.98x10^-06^ |
| 6 | rs11112007 | 12 | 104751554 | A | G | 0.41 | -0.6 | 1.10x10^-05^ |
| 7 | rs10513360 | 3 | 149834544 | T | T | 0.05 | -2 | 1.12x10^-05^ |
| 8 | rs12214745 | 6 | 121197242 | C | T | 0.02 | -16.62 | 1.61x10^-05^ |
| 9 | rs12071480 | 1 | 181483237 | A | A | 0.13 | -1.01 | 1.82x10^-05^ |
| 10 | rs114369201 | 5 | 171712979 | C | C | 0.02 | 1.87 | 1.84x10^-05^ |
| 11 | rs12450478 | 17 | 73737447 | G | A | 0.02 | -16.37 | 1.85x10^-05^ |
| 12 | rs12450609 | 17 | 73737432 | T | T | 0.02 | -16.37 | 1.87x10^-05^ |
| 13 | rs116947190 | 11 | 24211382 | GTGTA | A | 0.02 | 2.28 | 1.91x10^-05^ |
| 14 | rs117132178 | 18 | 57674362 | CTAT | T | 0.03 | -16.25 | 2.32x10^-05^ |
| 15 | rs156233 | 6 | 104872400 | A | T | 0.16 | -0.87 | 2.60x10^-05^ |
| 16 | rs1506230 | 18 | 62281281 | A | G | 0.02 | 1.72 | 2.76x10^-05^ |
| 17 | rs13097481 | 3 | 186700792 | G | C | 0.47 | 0.57 | 2.80x10^-05^ |
| 18 | rs74579032 | 6 | 38858179 | G | A | 0.03 | -16.25 | 3.14x10^-05^ |
| 19 | rs9314216 | 5 | 97289631 | CCAGCAGA | A | 0.04 | -2.19 | 3.21x10^-05^ |
| 20 | rs6707705 | 2 | 158966186 | C | A | 0.43 | 0.54 | 3.60x10^-05^ |
| 21 | rs1508213 | 16 | 65299309 | G | T | 0.07 | 0.97 | 3.69x10^-05^ |
| 22 | rs12082984 | 1 | 245101022 | CAT | T | 0.49 | 0.56 | 3.78x10^-05^ |
| 23 | rs1165640 | 3 | 193537991 | G | G | 0.09 | -1.24 | 3.92x10^-05^ |
| 24 | rs3887925 | 3 | 186665645 | A | G | 0.44 | -0.57 | 4.02x10^-05^ |
| 25 | rs17410050 | 1 | 4419997 | C | T | 0.42 | 0.56 | 4.04x10^-05^ |
| 26 | rs116917085 | 14 | 55844094 | C | T | 0.03 | 1.5 | 4.15x10^-05^ |
| 27 | rs35692099 | 11 | 118496524 | G | C | 0.04 | 1.17 | 4.36x10^-05^ |
| 28 | rs4391855 | 11 | 116350751 | AG | A | 0.04 | -2.18 | 4.45x10^-05^ |
| 29 | rs62068842 | 17 | 59917864 | T | A | 0.38 | 0.58 | 4.47x10^-05^ |
| 30 | rs6082312 | 20 | 21043102 | G | C | 0.50 | -0.55 | 4.48x10^-05^ |
